# Dynamic left main occlusion after aortic valve surgery: recognizing extrinsic compression by aortic root pseudoaneurysm—a case report

**DOI:** 10.1093/ehjcr/ytag600

**Published:** 2026-09-11

**Authors:** Sherif Seif, Babatunde Kukoyi, Abdalla Mujahid, Omar Nawaytou, Karim Ratib

**Affiliations:** Cardiology Department, Royal Stoke University Hospital, University Hospitals of North Midlands NHS Foundation Trust, Newcastle Rd, Stoke-on-Trent ST4 6QG, UK; Cardiology Department, Royal Stoke University Hospital, University Hospitals of North Midlands NHS Foundation Trust, Newcastle Rd, Stoke-on-Trent ST4 6QG, UK; Radiology Department, Royal Stoke University Hospital, University Hospitals of North Midlands NHS Foundation Trust, Newcastle Rd, Stoke-on-Trent ST4 6QG, UK; Liverpool Heart and Chest Hospital NHS Foundation Trust, Thomas Drive, Liverpool L14 3PE, UK; Cardiology Department, Royal Stoke University Hospital, University Hospitals of North Midlands NHS Foundation Trust, Newcastle Rd, Stoke-on-Trent ST4 6QG, UK

**Keywords:** Case report, Acute coronary syndrome, Left main coronary occlusion, Aortic root pseudoaneurysm, External coronary compression, Valve dehiscence

## Abstract

**Background:**

Extrinsic compression of the left main (LM) coronary artery by an aortic root pseudoaneurysm is an exceedingly rare but life-threatening cause of acute coronary syndrome following aortic valve surgery. Multimodality imaging is essential for diagnosis and surgical planning.

**Case summary:**

A 52-year-old male underwent mechanical aortic valve replacement and aortic root reconstruction for infective endocarditis complicated by a 23 mm aortic root abscess. Following initial recovery, he re-presented 2 months later with persistent chest pain on a background of 10 days of intermittent symptoms, with anterior-lateral ischaemic changes on electrocardiography (ECG). Computed tomography (CT) aortography excluded dissection but was not formally reported. In the catheterization laboratory, fluoroscopy revealed abnormal prosthetic valve rocking consistent with dehiscence. Coronary angiography demonstrated absent antegrade flow in the left anterior descending artery, impaired flow in the left circumflex artery, and a pulsatile, systole-predominant LM lesion strongly suggesting extrinsic compression. No percutaneous intervention was performed. Urgent formal CT review confirmed a 5.2 × 4.4 × 4.0 cm left ventricular outflow tract pseudoaneurysm with the LM coursing within its fold and apparent systolic occlusion. Serial ECGs showed gradual improvement, supporting a dynamic mechanism. After multidisciplinary review, the patient underwent successful composite graft replacement and pseudoaneurysm repair at a quaternary centre, with full recovery at 4-month follow-up.

**Discussion:**

This case illustrates three key lessons: the dynamic, systole-predominant angiographic hallmark of extrinsic LM compression distinguishes it from fixed atherosclerotic disease and prevents inappropriate percutaneous intervention; dynamic angiography and CT play complementary roles, and single-phase systolic CT may, in this setting, overestimate the severity of LM occlusion; and early referral to a specialist quaternary surgical centre is essential for definitive repair.

Learning points

1. **Not every ACS is a blocked artery.** A systole-worsening coronary lesion after aortic valve surgery means extrinsic compression, not atherosclerosis and avoid PCI.
2. **No single modality tells the whole story:** angiography reveals the dynamic compression; CT defines the anatomical cause and guides surgical planning.

## Introduction

Acute coronary syndrome (ACS) is most commonly caused by atherosclerotic plaque rupture, erosion, or calcified nodule with superimposed thrombus formation. Less frequent mechanisms include coronary embolism, spontaneous dissection, vasospasm, and extrinsic compression.^1^ Among post-surgical patients, compression of coronary arteries by an aortic root pseudoaneurysm is particularly uncommon but potentially fatal.

Aortic root pseudoaneurysm following valve replacement is a recognized, albeit rare, complication, occurring in approximately 0.2%–0.5% of cases and most frequently in the setting of infective endocarditis and aortic root abscess.^2^ Clinical presentations range from incidental detection to heart failure, aortic rupture, or ACS.^3^

We report a case of left main (LM) coronary occlusion caused by extrinsic compression from a large left ventricular outflow tract (LVOT) pseudoaneurysm in a patient who had recently undergone complex aortic root reconstruction for infective endocarditis. This case illustrates the importance of recognizing atypical angiographic features and the essential complementary roles of dynamic angiography and static computed tomography (CT) imaging.

## Summary figure

**Figure:**
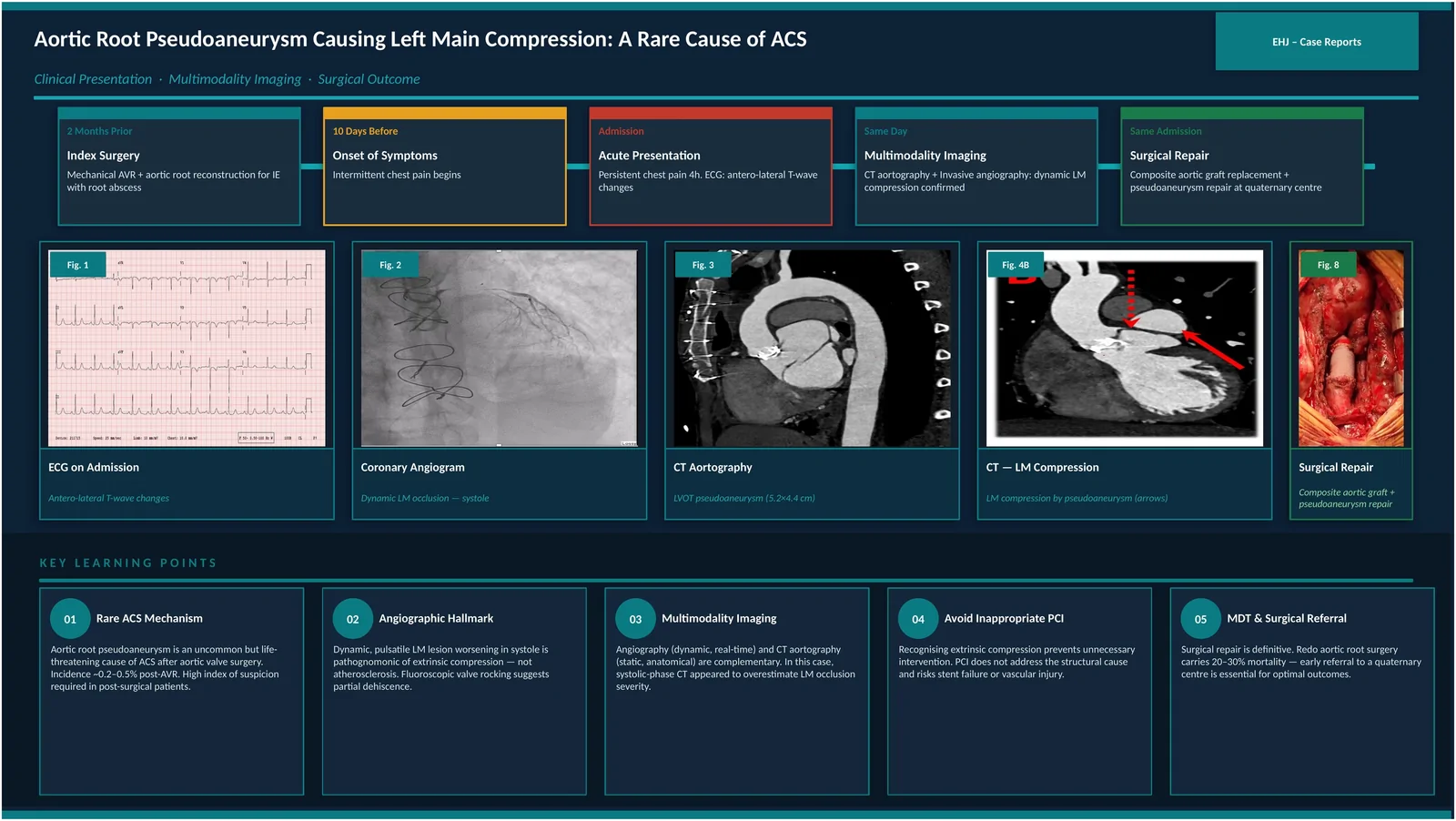


## Timeline

**Two months prior to presentation:** Mechanical aortic valve replacement and aortic root reconstruction for infective endocarditis complicated by aortic root abscess.

**Ten days before admission:** Onset of intermittent chest pain.

**Day of admission:** Chest pain became persistent (duration 4 h). Emergency transfer to a tertiary centre. Electrocardiography (ECG) demonstrated anterior-lateral ischaemic changes. Computed tomography angiography and urgent invasive coronary angiography performed.

**Same admission:** Multidisciplinary review. Transfer to quaternary aortic surgical centre for composite aortic graft replacement and pseudoaneurysm repair.

## Case presentation

A 52-year-old male was admitted with infective endocarditis complicated by an aortic root abscess. Pre-operative cardiac CT demonstrated an aortic root abscess measuring up to 23 mm, a coronary calcium score of zero, no significant coronary artery disease, and partially calcified aortic valve leaflets with appearances possibly representing vegetation (see Supplementary material online, *Figure S1*). He underwent mechanical aortic valve replacement and complex aortic root reconstruction, from which he made an initial satisfactory recovery and was discharged home.

Two months later, he re-presented by ambulance with a 4-h history of persistent chest pain, superimposed on a 10-day background of intermittent symptoms. He was therapeutically anticoagulated with warfarin (international normalized ratio 2.6). On admission, he appeared unwell but was haemodynamically stable. The 12-lead ECG demonstrated anterior-lateral T-wave inversion and ST-segment depression consistent with myocardial ischaemia (*Figure 1*).

**Figure 1:**
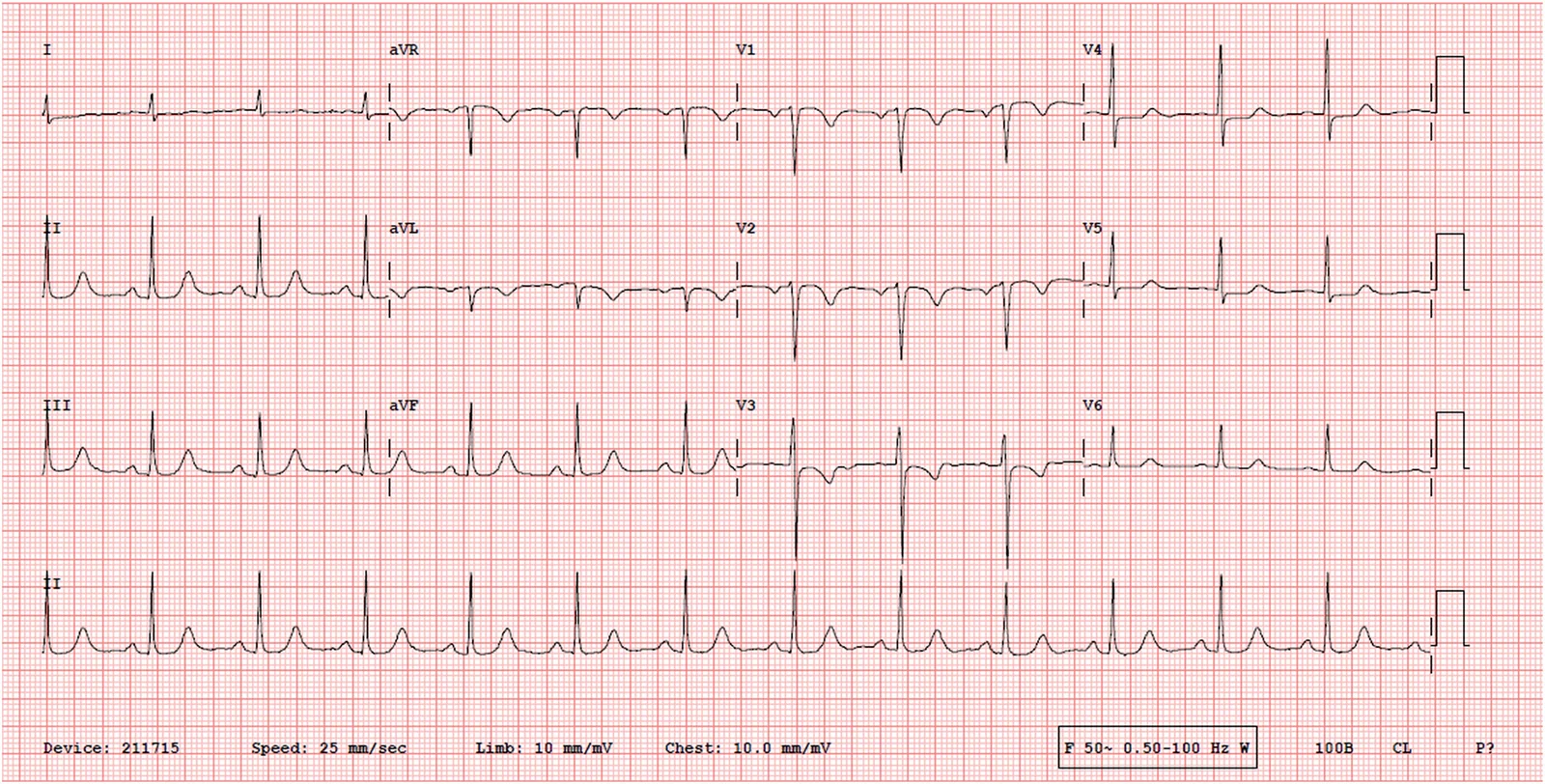
Twelve-lead electrocardiography recorded on admission demonstrating anterior-lateral T-wave inversion and ST-segment depression, consistent with myocardial ischaemia.

Given his recent aortic surgery, CT aortography was requested before coronary angiography to exclude aortic dissection. The CT was reported promptly by a radiology trainee, who confirmed the absence of dissection—the immediate clinical priority—but did not comment further on the aortic root, pending the final consultant report. At this stage, pseudoaneurysm was not clinically suspected, and the patient was cleared to proceed to the catheterization laboratory.

In the catheterization laboratory, fluoroscopy immediately demonstrated abnormal rocking movement of the mechanical aortic prosthesis, raising concern for valve dehiscence (see Supplementary material online, *Video S1*). Coronary angiography revealed absent antegrade flow to the left anterior descending artery (LAD) and significantly impaired flow to the left circumflex artery. Notably, the LM stem lesion exhibited a dynamic, pulsatile character that worsened markedly in systole, a behaviour that did not fit conventional atherosclerotic disease and aroused strong suspicion of extrinsic compression (*Figure 2*; Supplementary material online, *Video S2*). The right coronary artery was widely patent and provided retrograde collateral filling of the LAD. Given the absence of coronary artery disease on the pre-operative CT (coronary calcium score of zero), this well-developed collateral network is best explained by gradual, progressive extrinsic compression of the LM and proximal LAD by the slowly enlarging pseudoaneurysm over the 2-month interval since surgery rather than by an acute occlusive event (see Supplementary material online, *Video S3*). No percutaneous intervention was performed at this stage.

**Figure 2:**
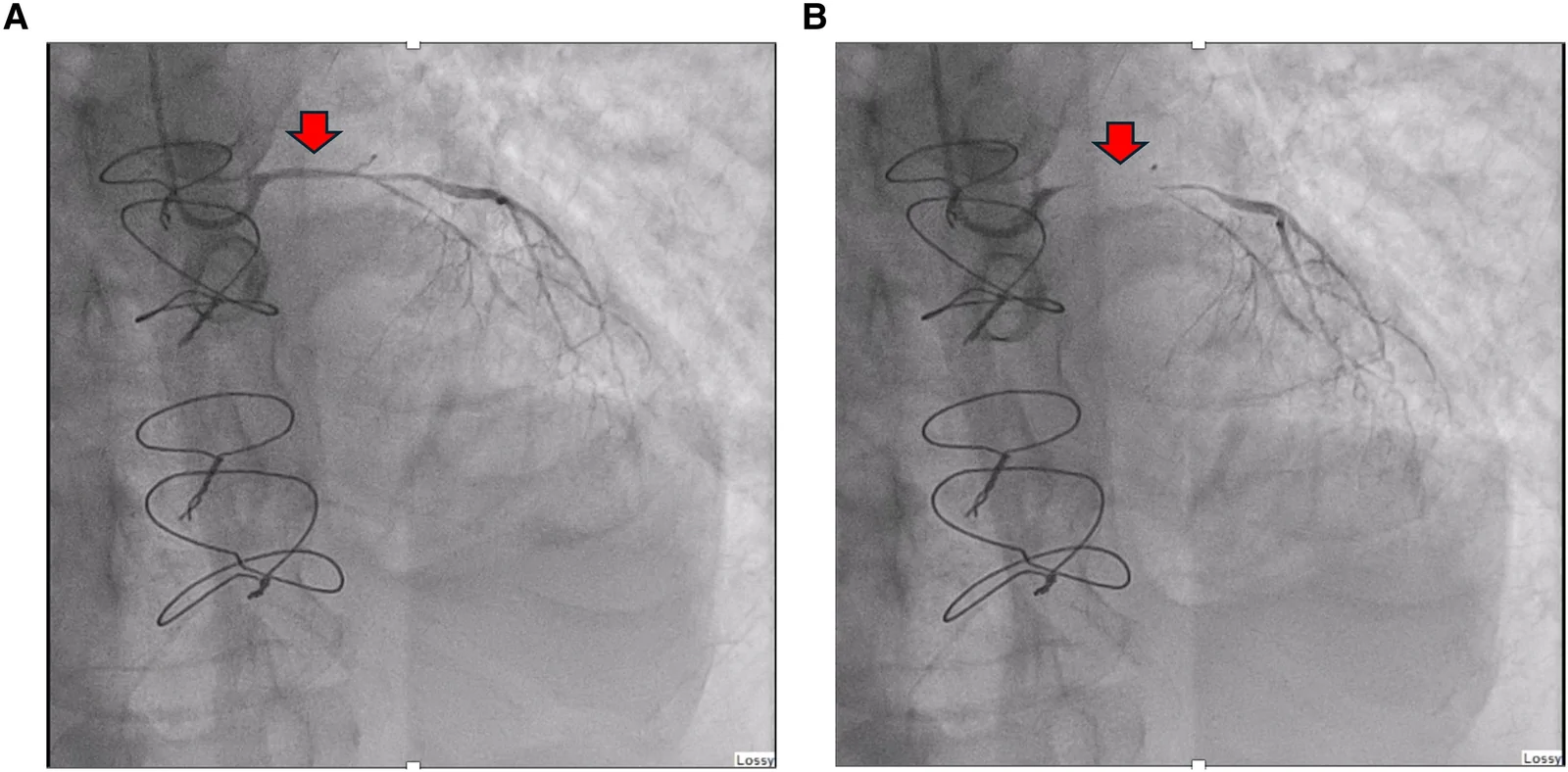
Coronary angiogram demonstrating dynamic, pulsatile occlusion of the left main stem and left circumflex artery (red arrows), varying with the cardiac cycle: (*A*) diastole, partial filling preserved; (*B*) systole, near-complete occlusion, consistent with extrinsic compression.

In light of these unexpected angiographic findings, the same CT dataset was urgently re-reviewed by the consultant radiologist. On formal reporting—prompted by the angiographic suspicion of extrinsic compression—a large pseudoaneurysm measuring 5.2 × 4.4 × 4.0 cm was identified, arising from the LVOT immediately beneath the aortic prosthesis and extending postero-superiorly, which is consistent with partial dehiscence at the surgical repair site (*Figure 3*). The LM origin was seen coursing within a fold of the pseudoaneurysm, with apparent occlusion on the systolic-phase acquisition (*Figure 4*). This confirmed the operator’s suspicion: the LM was being compressed extrinsically by the expanding pseudoaneurysm, not occluded by fixed coronary disease. Serial ECG monitoring demonstrated gradual improvement in ischaemic changes, and the patient’s symptoms partially settled, further supporting a dynamic rather than fixed mechanism.

**Figure 3:**
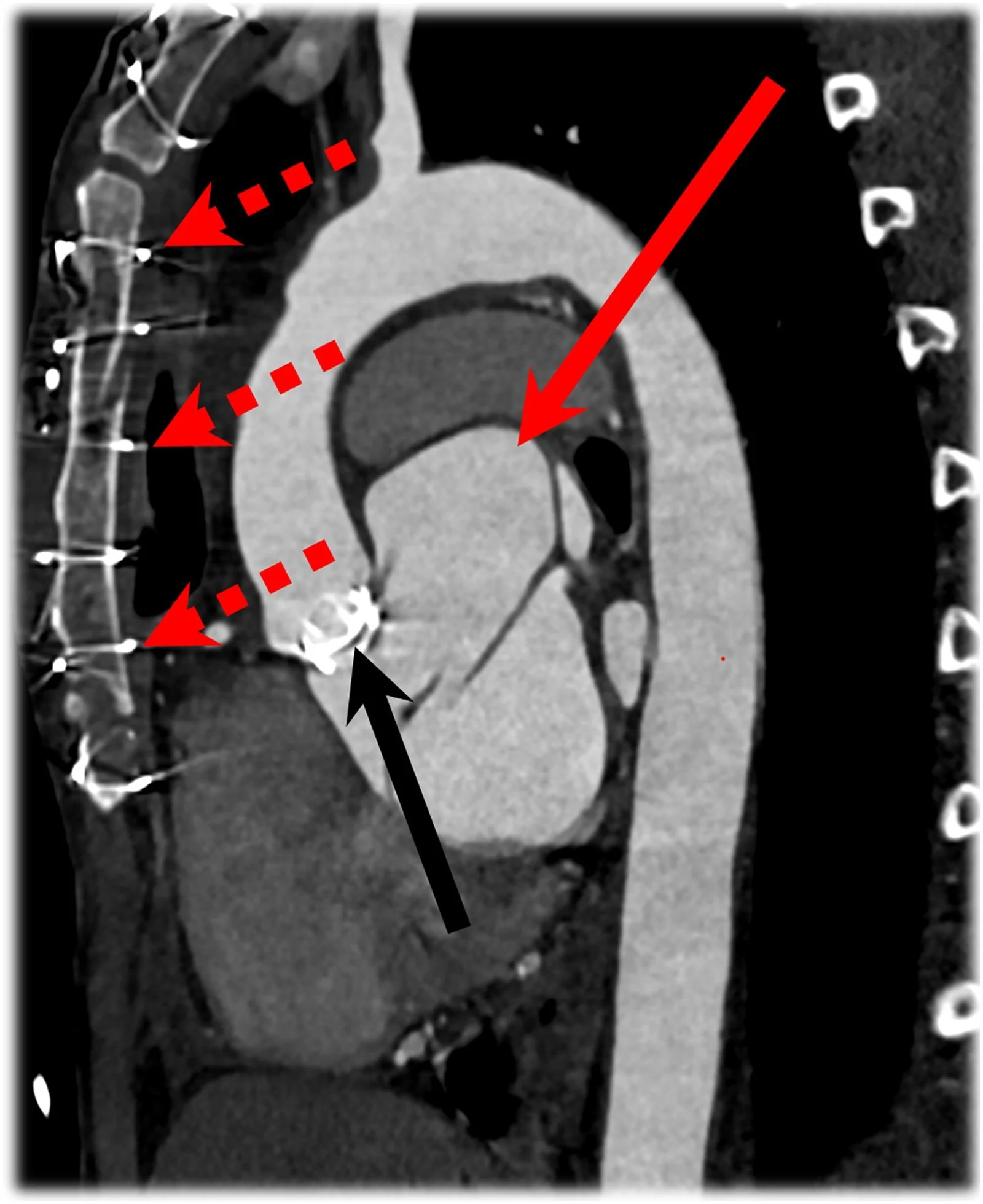
Obliqued sagittal computed tomography angiogram demonstrating a large post-surgical pseudoaneurysm (solid red arrow) arising from the left ventricular outflow tract, immediately inferior to the mechanical aortic valve (solid black arrow). Median sternotomy wires are seen *in situ* (dashed red arrows).

**Figure 4:**
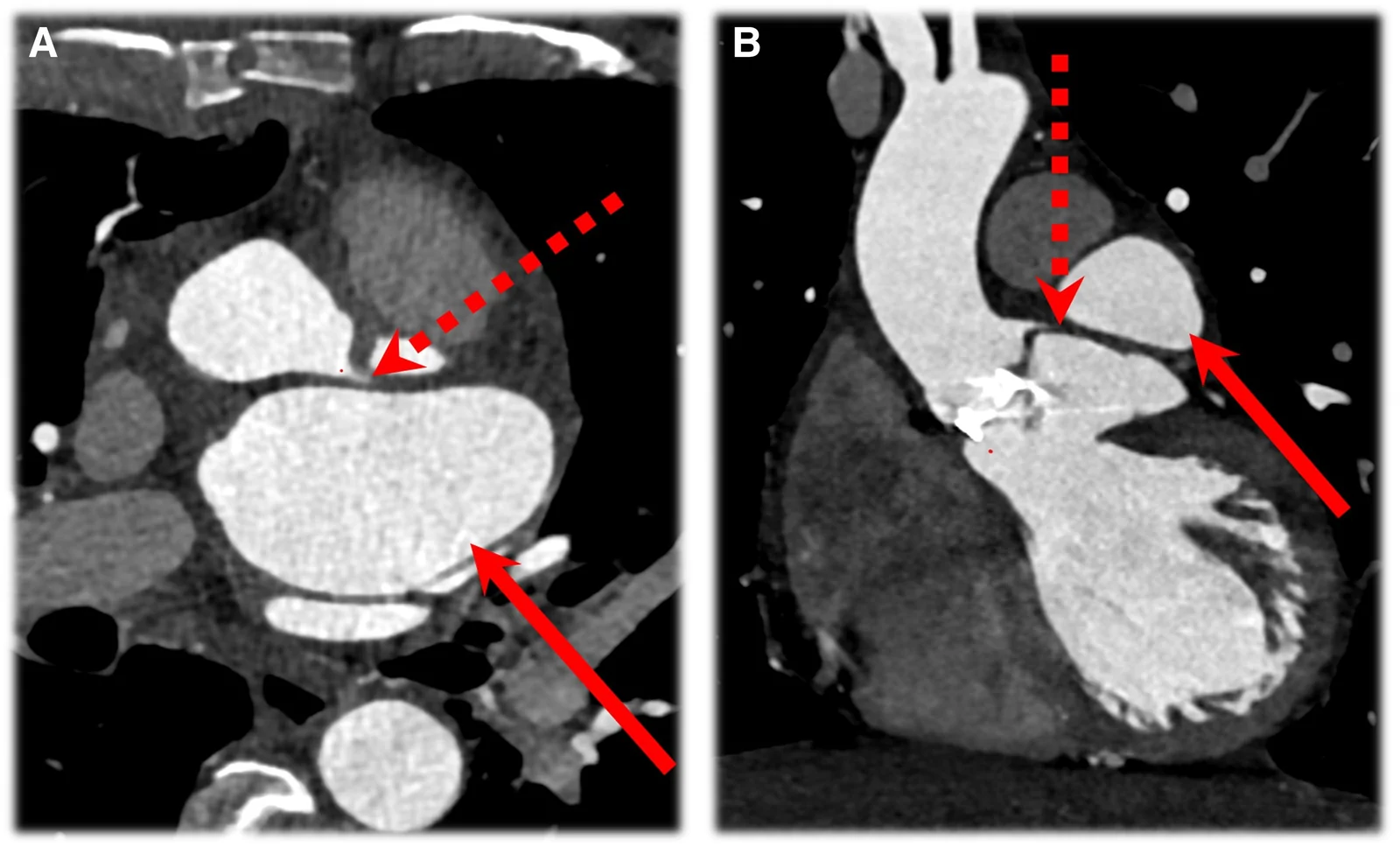
Axial (*A*) and coronal oblique (*B*) computed tomography angiogram images demonstrating occlusion of the left main stem coronary artery (dashed arrows) caused by extrinsic compression from the adjacent pseudoaneurysm (solid arrows).

The case was discussed urgently with the original surgical team, who assessed redo surgery as carrying prohibitively high operative risk given the complexity of the prior reconstruction. The patient was transferred the next day to a quaternary aortic surgical centre, where he underwent successful composite aortic graft replacement and repair of the LVOT pseudoaneurysm (*Figure 5*; Supplementary material online, *Video S4*). His postoperative course was uneventful, and he was discharged in good condition.

**Figure 5:**
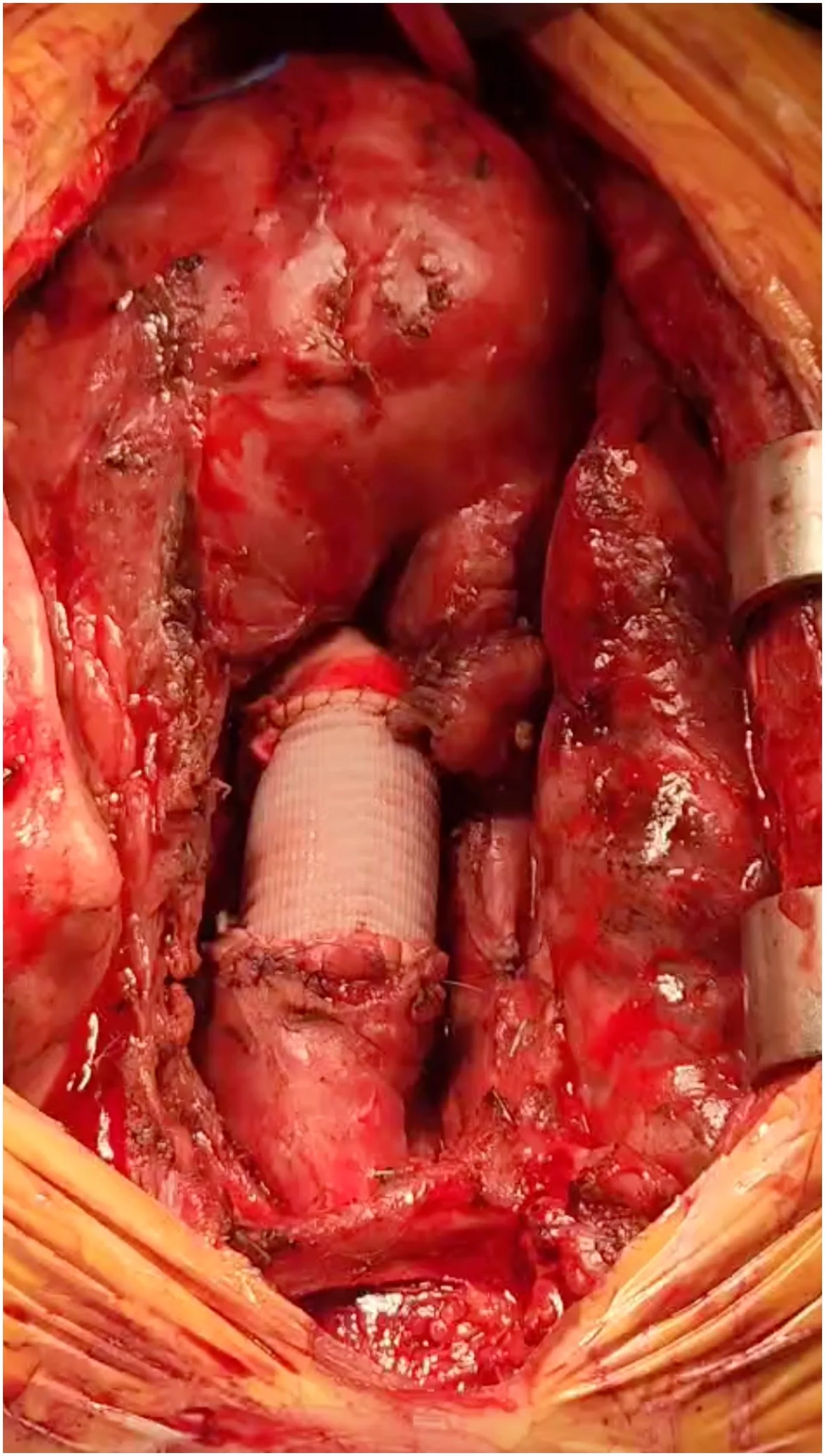
Intra-operative photograph showing the final result of composite aortic graft replacement.

At outpatient review 4 months later, the patient was doing well. Sternal healing was satisfactory, and he reported no recurrence of chest pain, no exertional symptoms, and no features of heart failure. He remained on anticoagulation and was under regular cardiology follow-up.

## Discussion

Aortic root pseudoaneurysm is a recognized but uncommon complication following aortic valve replacement, reported in approximately 0.2%–0.5% of cases.^2^ It occurs most frequently in the context of infective endocarditis with aortic root abscess, where tissue destruction and inflammation compromise the integrity of the surgical repair site. Though often detected incidentally or through heart failure symptoms, pseudoaneurysm expansion can compress adjacent coronary arteries—particularly the left main stem, given its anatomical proximity to the aortic root—leading to life-threatening ACS. Mortality from untreated aortic root pseudoaneurysm is high, and reoperative surgery itself carries perioperative mortality reported to exceed 20%–30% in some series.^4^

This case highlights several clinical lessons. First, the dynamic, pulsatile morphology of the LM lesion on angiography—worse in systole and absent in fixed atherosclerotic stenosis—is the key angiographic hallmark of extrinsic coronary compression. Recognition of this pattern is essential to avoid inappropriate percutaneous coronary intervention (PCI), which carries a risk of vascular injury and stent failure in this context and does not address the underlying structural cause.^4,5^

Second, dynamic angiography and CT played complementary roles: angiography provided real-time evidence of pulsatile compression, while CT aortography defined the anatomical extent of the pseudoaneurysm and its relationship to the LM origin, enabling surgical planning. In our patient, the systolic-phase CT acquisition appeared to overestimate the severity of LM occlusion relative to the dynamic angiographic findings; whether this is a broader phenomenon in extrinsic coronary compression merits further evaluation.

Third, surgical repair remains the definitive treatment for aortic root pseudoaneurysm with coronary compromise.^2,3^ The decision to refer to a specialized quaternary centre and avoid percutaneous intervention reflects optimal multidisciplinary management in this complex scenario.

Several similar cases of LM compression by aortic root pseudoaneurysm have been reported. Ezeh *et al*.^6^ described a closely analogous presentation following prosthetic valve endocarditis, in which the patient also required redo surgery and coronary bypass grafting, while Molina-Lopez *et al*.^7^ reported extrinsic LM compression from a left sinus of Valsalva pseudoaneurysm after undiagnosed endocarditis, where the diagnosis was similarly established through coronary angiography and computed tomography angiography. Nerla *et al*.^8^ and Neupane *et al*.^9^ further described dynamic LM compression; notably, Neupane’s patient required salvage intravascular ultrasound-guided PCI as a bridge to surgery when haemodynamic compromise precluded deferral of intervention, in contrast to our case where the clinical stability allowed PCI to be deliberately avoided. Niclauss *et al*.^5^ and Lacalzada-Almeida *et al*.^10^ reported coronary compression by a pseudoaneurysm following infective endocarditis.^5,10^ A systematic review of ascending aortic pseudoaneurysm interventions^11^ further underscores the rarity and complexity of this diagnosis. Compared with these reports, our case is distinguished by the diagnostic sequence: fluoroscopy first raised suspicion through visible prosthetic valve rocking, angiography then confirmed dynamic systole-predominant LM compression, and PCI was deliberately avoided at the index procedure itself—followed by urgent multimodality CT correlation that established the underlying structural cause.

## Conclusion

Aortic root pseudoaneurysm should be considered as a rare but life-threatening cause of ACS in patients with prior aortic valve surgery. Recognition of characteristic dynamic angiographic features and the use of complementary multimodality imaging are critical to establishing the correct diagnosis, avoiding inappropriate intervention, and facilitating timely surgical referral.

## Supplementary Material

ytag600_Supplementary_Data

## Data Availability

Data sharing is not applicable to this article as no datasets were generated or analysed during the preparation of this work.

## Lead author biography

Dr Sherif Seif is one of the interventional cardiologists at Royal Stoke University Hospital in the UK. He holds MBBCh, MSc, and MD degrees and is a Member of the Royal College of Physicians (MRCP, UK). His clinical and research interests include complex coronary intervention, acute coronary syndromes, and structural heart disease. He has contributed to case reports and clinical publications in cardiovascular medicine.

## Notes

**Consent:** The authors confirm that written consent for submission and publication of this case report, including all accompanying images, videos, and associated text, has been obtained from the patient, in line with COPE guidance.
